# Modulating Regional Motor Cortical Excitability with Noninvasive Brain Stimulation Results in Neurochemical Changes in Bilateral Motor Cortices

**DOI:** 10.1523/JNEUROSCI.2853-17.2018

**Published:** 2018-08-15

**Authors:** Velicia Bachtiar, Ainslie Johnstone, Adam Berrington, Clark Lemke, Heidi Johansen-Berg, Uzay Emir, Charlotte J. Stagg

**Affiliations:** ^1^Nuffield Department of Clinical Neurosciences, Functional Magnetic Resonance Imaging of the Brain (FMRIB), Wellcome Centre for Integrative Neuroimaging, University of Oxford, Oxford, OX3 9DU, United Kingdom, and; ^2^Department of Psychiatry, Oxford Centre for Human Brain Activity, Wellcome Centre for Integrative Neuroimaging, University of Oxford, Oxford, OX3 7JX, United Kingdom

**Keywords:** DTI, GABA, M1, MRS, plasticity, tDCS

## Abstract

Learning a novel motor skill is dependent both on regional changes within the primary motor cortex (M1) contralateral to the active hand and also on modulation between and within anatomically distant but functionally connected brain regions. Interregional changes are particularly important in functional recovery after stroke, when critical plastic changes underpinning behavioral improvements are observed in both ipsilesional and contralesional M1s. It is increasingly understood that reduction in GABA in the contralateral M1 is necessary to allow learning of a motor task. However, the physiological mechanisms underpinning plasticity within other brain regions, most importantly the ipsilateral M1, are not well understood. Here, we used concurrent two-voxel magnetic resonance spectroscopy to simultaneously quantify changes in neurochemicals within left and right M1s in healthy humans of both sexes in response to transcranial direct current stimulation (tDCS) applied to left M1. We demonstrated a decrease in GABA in both the stimulated (left) and nonstimulated (right) M1 after anodal tDCS, whereas a decrease in GABA was only observed in nonstimulated M1 after cathodal stimulation. This GABA decrease in the nonstimulated M1 during cathodal tDCS was negatively correlated with microstructure of M1:M1 callosal fibers, as quantified by diffusion MRI, suggesting that structural features of these fibers may mediate GABA decrease in the unstimulated region. We found no significant changes in glutamate. Together, these findings shed light on the interactions between the two major network nodes underpinning motor plasticity, offering a potential framework from which to optimize future interventions to improve motor function after stroke.

**SIGNIFICANCE STATEMENT** Learning of new motor skills depends on modulation both within and between brain regions. Here, we use a novel two-voxel magnetic resonance spectroscopy approach to quantify GABA and glutamate changes concurrently within the left and right primary motor cortex (M1) during three commonly used transcranial direct current stimulation montages: anodal, cathodal, and bilateral. We also examined how the neurochemical changes in the unstimulated hemisphere were related to white matter microstructure between the two M1s. Our results provide insights into the neurochemical changes underlying motor plasticity and may therefore assist in the development of further adjunct therapies.

## Introduction

It has been shown consistently that plasticity in the motor system, whether underlying the learning of novel motor skills or their relearning after stroke, involves changes within and between a network of anatomically distributed motor regions. In particular, it is clear from studies examining the neural control of unilateral motor behaviors that the interaction between the two primary motor cortices (M1s) is vital for motor learning (Perez and Cohen, 2009; Reis et al., 2009; Di Pino et al., 2014). However, little is known about exactly how this interaction occurs and what physiological changes in distant nodes underpin interregional plasticity. Animal and computational models have suggested that network-level plasticity may depend on the relationship between high-frequency oscillations within the network-nodes, which are driven at least in part by regional GABAergic activity (Cabral et al., 2011; Hall et al., 2011; Nowak et al., 2017). It is plausible to hypothesize, therefore, that decreases in GABA in distant network nodes are necessary to increase coherence, change connectivity, and therefore allow network-level plasticity to occur. Here, we tested this hypothesis by studying neurochemical changes underpinning plasticity in both M1s simultaneously using transcranial direct current stimulation (tDCS).

Anodal tDCS to M1 is thought to induce long-term potentiation (LTP)-like changes within the stimulated region (Hess et al., 1996; Fritsch et al., 2010; Stagg and Nitsche, 2011; Monte-Silva et al., 2013) and has been demonstrated to affect regional neurochemistry in a manner similar to motor learning, most notably resulting in a regional decrease in GABA concentration (Floyer-Lea et al., 2006; Stagg et al., 2009, 2011a; Stagg and Nitsche, 2011; Bachtiar et al., 2015).

The neurochemical changes due to tDCS are reflected in behavioral changes, with anodal tDCS improving learning if applied during practice of a motor task (Nitsche et al., 2003; Galea et al., 2011; Stagg et al., 2011a; Cuypers et al., 2013; Kim et al., 2014; Sriraman et al., 2014; Amadi et al., 2015; Waters et al., 2017). This beneficial effect of anodal tDCS on motor learning has led to tDCS being suggested as a putative adjunct therapy to enhance motor rehabilitation after stroke, with some success in small proof-of-principle studies (Fregni et al., 2005; Hummel and Cohen, 2005; Hummel et al., 2005; Kim et al., 2006; Boggio et al., 2007; Lindenberg et al., 2010; Allman et al., 2016).

In addition to applying anodal tDCS to the ipsilesional hemisphere after stroke, some studies have shown that applying cathodal tDCS to the contralesional hemisphere to reduce local activity here also leads to behavioral improvements in the paretic hand. This functional improvement is accompanied by increased activity in the ipsilesional M1, consistent with the hypothesis of interhemispheric imbalance in this patient group (Fregni et al., 2005; Boggio et al., 2007; Kim et al., 2010; Stagg et al., 2012; O'Shea et al., 2014). However, the mechanism by which contralesional tDCS can increase activity in the contralateral and ipsilesional M1 is not understood.

In this study, we used a novel two-voxel magnetic resonance spectroscopy (MRS) approach to quantify GABA and glutamate changes concurrently within the left and right M1 during the three most commonly used tDCS montages: anodal to left M1, cathodal to left M1, and bilateral, in which electrodes are placed over both M1s. We also examined how the neurochemical changes in the unstimulated hemisphere were related to white matter microstructure between the two M1s.

## Materials and Methods

### Participants

Twelve healthy participants (two male, aged 20–32 years, mean 25 years) gave their informed consent to participate in this study in accordance with ethical approval from the East London Research Ethics Committee (reference #10/H0703/50). All were right-handed as assessed by the Edinburgh Handedness Inventory (Oldfield, 1971).

### Experimental design

**Figure 1.**
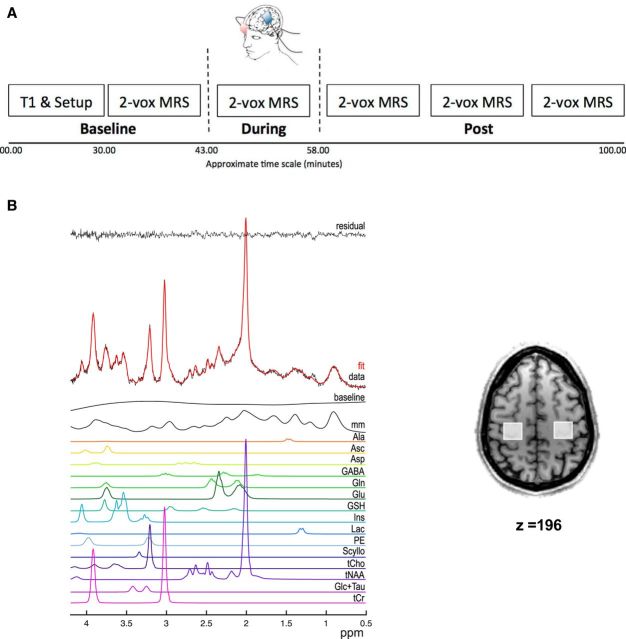
***A***, Schematic outlining the protocol of the 7 T MRS sessions. ***B***, Representative spectrum from one acquisition block from one voxel, as analyzed by LCModel, showing the raw data (red) as well as the individual metabolite fits (colored lines) and the residual noise (black). Inset shows the location of both voxels from which MRS was acquired.

Participants undertook five experimental sessions: four of which were MRS scans at 7 T to assess changes in GABA and glutamate concentrations in both M1s simultaneously due to sham, anodal, or cathodal tDCS applied to the left M1 or to bilateral tDCS with the anode positioned over left M1 (see Fig. 1 for an experimental outline).

To relate the magnitude of any changes in neurochemicals across anatomically distant regions, we additionally acquired diffusion tensor imaging (DTI) measures of white matter microstructure in a fifth imaging session performed at 3 T.

All MRS-tDCS sessions were separated by at least 1 week and the order of the sessions was counterbalanced across the group. Two subjects did not complete the bilateral MRS-tDCS session due to personal time constraints. In each MRS-tDCS session, MRS spectra were acquired over five time points: at baseline, during tDCS, and at three time points after tDCS: Post 1 = ∼1–13 min after tDCS, Post 2 = ∼14–26 min after tDCS, and Post 3 = ∼27–39 min after tDCS (see Figure 1 for experimental outline). Subjects watched a nature documentary for the duration of the experiment.

#### tDCS

A DC stimulator (Magstim) delivered a 1 mA current to the brain via electrodes measuring 5 × 7 cm (Easycap). For the anodal, cathodal, and sham sessions, one electrode was centered over the left M1 positioned 5 cm lateral to Cz and the other over the contralateral supraorbital ridge. For the bilateral session, the anode was placed over the left M1 and the cathode over the right M1, each electrode positioned 5 cm lateral to Cz. Although this method of electrode placement does not take into account individual's head sizes, we believe that differences in electrode placement between individuals are likely to be relatively minor.

High-chloride electrolyte gel (Easycap) was used as the conducting medium between the scalp and electrodes. The electrodes contained 5 kΩ resistors and extension leads connected the stimulator, which was located outside of the magnetic field, to the subject positioned in the scanner. For the real stimulation conditions (anodal, cathodal, bilateral) the current was ramped up over 10 s and was then held at 1 mA for 10 min before being ramped down over 10 s. For sham stimulation, the DC stimulator was ramped up for 10 s and then switched off, as described previously (Stagg et al., 2011b).

#### MRS data acquisition

For full details of the MRS acquisition approach, see Lemke et al. (2015). Briefly, MRS data were acquired on a 7 T Siemens MR system with a 32-channel receive array head-coil. T_1_-weighted images (MPRAGE, 192 × 1 mm axial slices, TR/TE = 2200/2.82 ms, flip-angle = 7○, FOV 192 × 100) were used to place a 2 × 2 × 2 cm voxel of interest over the left and right precentral knobs, a known landmark for hand motor representation (Yousry et al., 1997).

Spectra were measured using the sLASER sequence (TR/TE = 7000/30 ms) (Oz and Tkáč, 2011) with VAPOR water suppression (Tkáč et al., 1999). Spectra were acquired in an interleaved fashion from the two voxels (64 transients each) (Lemke et al., 2015). Each block of MRS data acquisition took ∼12 min.

#### MRI acquisition

MRI data were acquired using a 3 T Siemens Verio MRI System and a 32-channel receive head coil. A whole-brain high-resolution structural image (1.0 mm isotropic) was also collected using a multigradient echo sequence to enable anatomic registration (TE1 = 1.79 ms, TE2 = 3.65 ms, TE3 = 5.51 ms, TE4 = 7.37 ms, TR = 2530 ms, FOV = 256 × 256).

To relate the microstructure of the corpus callosum (CC) to the change in MRS metrics acquired at 7 T, echoplanar diffusion weighted imaging was acquired (diffusion directions = 64, b-value = 1500 s/mm^2^, voxel dimensions = 2 × 2 × 2 mm, 64 slices, TR = 8900 ms, TE = 91 ms).

We also acquired fMRI scans to enable functional localization of M1 in each subject. A total of 130 echoplanar volumes were collected (3 mm isotropic voxels, TE = 30 ms, TR = 3000 ms, FOV = 192 × 192 mm) while the participant performed visually cued hand tapping at 1 Hz with either the left or right hand, interspersed with blocks of rest. Blocks of movement and rest were 30 s long.

### Data analysis

#### MRS analysis

As has been described previously (Lemke et al., 2015), standard preprocessing, including eddy current correction and zero-order phasing of array coil spectra, was performed using in-house scripts. Any residual water signal was removed from the metabolite spectra using Hankel–Lanczos singular value decomposition (Cabanes et al., 2001). Neurochemicals were quantified using LCModel analysis (RRID:SCR_014455) (Provencher, 2001). The following criteria were applied for excluding spectra with poor quality: (1) Cramer–Rao lower bounds > 50%, (2) water linewidths at full width at half-maximum (FWHM) >15 Hz, and (3) signal-to-noise ratio <10. Of the 460 spectral blocks acquired (five time points from two voxels in 12 subjects with three to four sessions per subject), 15 blocks from five subjects were excluded on these criteria for the GABA measurements and two spectra from one subject were excluded for the glutamate measurement.

As is common, neurochemical concentrations are presented here as ratios relative to total creatine (tCr) (Soares and Law, 2009; Mullins et al., 2014). To examine the relationship between individuals' baseline GABA levels and MRI measures, the mean baseline GABA:Cr value for right and left M1 in each subject across the sessions was calculated.

#### fMRI analysis

Analysis was performed using FEAT (FMRI Expert Analysis Tool) version 6.00, part of FSL (FMRIB's Software Library, www.fmrib.ox.ac.uk/fsl, RRID:SCR_002823) (Smith et al., 2004; Woolrich et al., 2009; Jenkinson et al., 2012). Registration to high-resolution structural T1-weighted images was performed using FLIRT. Registration from structural images to standard space was then further refined using FNIRT nonlinear registration (Andersson et al., 2007a,b).

The following prestatistics processing was then applied: motion correction using MCFLIRT (Jenkinson et al., 2002), nonbrain removal using BET (Smith, 2002; Brain Extraction Tool, RRID:SCR_014586), spatial smoothing using a Gaussian kernel of FWHM 5 mm, grand-mean intensity normalization for the entire 4D dataset by a single multiplicative factor, and high-pass temporal filtering (Gaussian-weighted least-squares straight line fitting with σ = 57.0s). Independent component analysis-based exploratory analysis was performed using MELODIC (Beckmann et al., 2005) to investigate the possible presence of unexpected artifacts or activation. FSL Motion Outliers was run on all functional scans to identify any time points corrupted by large motion. The resulting confound matrix was then included in the general linear model to remove the effects of these time points on the resulting analyses.

For each participant, contrasts of left press > rest and right press > rest were performed with a *Z* threshold of 2.3 and cluster *P* threshold of 0.05. Group-level analysis was then performed to find group average clusters of activation (*Z* threshold = 2.3, cluster *P* threshold = 0.05). These group-level clusters were then transformed into individuals' DTI space using FLIRT (Andersson et al., 2007a,b) and were used as functionally defined M1 masks.

#### DTI analysis

Diffusion data were processed using tools from FSL (Smith et al., 2004; Jenkinson et al., 2012). Diffusion-weighted scans were first corrected for eddy-current distortions using eddycorrect and then brain extracted using BET. Local diffusion tensors were then fit using the dtifit command in FSL. Markov Chain Monte Carlo sampling was then used to build the diffusion orientation distribution functions for each voxel (Behrens et al., 2007) using Bedpostx (BEDPOSTX; FMRIB; Behrens et al., 2003, 2007), modeling a total of up to two fibers per voxel and using total of 1000 iterations.

##### Tractography.

Tracts running from the CC to the motor cortices were estimated using PROBTRACKX2 (Behrens et al., 2003, 2007). A mask of the CC was created using the Juelich thr25 atlas and transformed into each participant's DTI space. Bilateral M1s were functionally defined during the same 3 T scan. The CC mask was used as the seed mask, with functionally defined bilateral M1s as a one-way waypoint, meaning that only pathways that passed through the CC and reached at least M1 would be retained. A total of 5000 individual pathways were generated from each voxel within the seed mask using a step length of 0.5 mm and a maximum of 2000 steps. A cosine curvature threshold of 0.2 (∼80°) was used to limit how sharply pathways could deflect during tract generation. This procedure generated a tract map for each participant in which voxel values represented the total number of pathways passing through each brain voxel.

For each participant, this tract map was thresholded at >7500, binarized, and then the overlap between this mask and the CC mask was used to define a M1–M1 CC mask. The mean fractional anisotropy (FA) value across all voxels within this mask was then extracted and used in subsequent correlation analyses.

A first control region, the area of the CC carrying tracts connecting left M1 to right somatosensory cortex was defined using the method described above, but using Harvard–Oxford defined right somatosensory cortex and functional defined left M1 as waypoints in PROBTRACKX2. This tract was thresholded to <25 and masked with the Harvard–Oxford CC mask. A second control region in right corticospinal tract (rCST) was defined by using the right M1 as a seed point and right internal capsule as a waypoint, with the same parameters as for the M1–M1 tract. For each participant, this tract was thresholded at >2000, binarized, and then the region of interest was defined as the area within this tract mask falling within a 25 × 25 × 25 voxel volume around the center of the internal capsule. This approach was taken to ensure that the volume of the rCST mask and the M1–M1 CC mask were not significantly different (paired *t* test, *t*_(11)_ = 1.749, *p* = 0.11).

## Results

We first wanted to test whether there were any baseline differences in neurochemicals across sessions. We therefore performed a repeated-measures ANOVA (RM-ANOVA) with within-subject factors of stimulation condition (anodal, cathodal, bilateral, sham), hemisphere (right M1, left M1), and neurochemical (GABA, Glu). This demonstrated no significant main effect of stimulation condition (*F*_(3,21)_ = 0.928, *p* = 0.445), no stimulation by hemisphere interaction (*F*_(3,21)_ = 2.357, *p* = 0.101), and no significant stimulation by neurochemical interaction (*F*_(3,21)_ = 2.515, *p* = 0.086).

Therefore, to control for intersubject differences in baseline neurochemical concentration, we calculated the change in each neurochemical from baseline at each time point for all subjects and sessions and used these values in all subsequent analyses.

All neurochemical concentrations presented here are referenced to tCr (Cr + PCr = tCr). We therefore wished to test for any changes in creatine over time that might potentially affect our results. We found no change in tCr over time in either M1 (RM-ANOVA for each M1 separately with one factor of stimulation condition (anodal, cathodal, bilateral, sham) and one factor of time (baseline, during, Post 1, Post 2, Post 3); no main effect of stimulation or time, or stimulation by time interaction for either M1 (all *p* > 0.05).

### tDCS to the left M1 leads to significant changes in neurochemicals in both hemispheres

We wished to investigate whether there were any significant changes in neurochemicals in response to tDCS. A RM-ANOVA with within-subjects factors of stimulation condition (anodal, cathodal, bilateral, sham), hemisphere (right M1, left M1), neurochemical (glutamate, GABA), and time (during, Post 1, Post 2, Post 3) demonstrated a significant main effect of stimulation condition (*F*_(3,15)_ = 5.235, *p* = 0.011); no significant main effect of hemisphere (*F*_(1,5)_ = 0.006, *p* = 0.940); no main effect of neurochemical (*F*_(1,5)_ = 0.890, *p* = 0.890); and no significant main effect of time (*F*_(3,15)_ = 0.904, *p* = 0.463). Importantly, we demonstrated a significant interaction between stimulation condition and neurochemical (*F*_(3,15)_ = 4.223, *p* = 0.024) (see Table 1 for full results).

**Table 1.** Full results of RM-ANOVA with one factor of stimulation condition (anodal, cathodal, bilateral, sham), one factor of hemisphere (right M1, left M1), one factor of neurochemical (glutamate, GABA), and one factor of time (during, Post 1, Post 2, Post 3)

Effect	df	*F*	*p*	η^2^
Stimulation condition	3	5.235	0.011	0.511
Error (stimulation condition)	15			
Hemisphere	1	0.006	0.940	0.001
Error (hemisphere)	5			
neurochemical	1	0.890	0.389	0.151
Error (neurochemical)	5			
Time	3	0.904	0.462	0.153
Error (time)	15			
Stimulation condition*hemisphere	3	0.538	0.663	0.097
Error (stimulation condition * hemisphere)	15			
Stimulation condition * neurochemical	3	2.101	0.024	0.458
Error (stimulation condition * neurochemical)	15			
Hemisphere* neurochemical	1	0.002	0.969	<0.001
Error (hemisphere * neurochemical)	5			
Stimulation condition * time	9	1.648	0.130	0.248
Error (stimulation condition * time)	45			
Hemisphere* time	3	1.120	0.372	0.183
Error (hemisphere * time)	15			
Time* neurochemical	3	0.047	0.618	0.109
Error (time * neurochemical)	15			
Stimulation condition *hemisphere* neurochemical	3	0.550	0.407	0.171
Error (stimulation condition * hemisphere *neurochemical)	15			
Stimulation condition *hemisphere* time	9	0.306	0.969	0.058
Error (stimulation condition * hemisphere * time)	45			
Hemisphere * neurochemical * time	3	0.787	0.520	0.136
Error (stimulation condition * neurochemical * time)	15			
Stimulation condition * neurochemical * time	9	0.885	0.545	0.150
Error (stimulation condition * neurochemical * time)	45			
Stimulation condition * neurochemical * hemisphere * time	9	1.671	0.125	0.250
Error (stimulation condition neurochemical * hemisphere * time)	45			

To explore the effects of the different stimulation conditions further, we investigated whether there were significant changes in the left and right M1s due to tDCS when the neurochemicals and hemispheres were considered separately using RM-ANOVAs and the within-subjects factors of stimulation condition (anodal, cathodal, bilateral, sham) and time (during, Post 1, Post 2, Post 3). We found a significant main effect of stimulation condition on the change in GABA concentration within both left and right M1s (left M1, GABA: *F*_(3,24)_ = 4.739, *p* = 0.016; right M1, GABA: *F*_(3,24)_ = 7.513, *p* = 0.001). There was no significant main effect of time (left M1, GABA: *F*_(3,15)_ = 2.755, *p* = 0.079; right M1, GABA: *F*_(3,24)_ = 0.030, *p* = 0.993) and no interaction between stimulation and time (left M1, GABA: *F*_(9,45)_; right M1, GABA: *F*_(9,72)_ = 1.311, *p* = 0.246) in either hemisphere.

However, tDCS had no significant effect of stimulation on glutamate concentration within either hemisphere (left M1, glutamate: main effect of stimulation condition: *F*_(3,27)_ = 2.110, *p* = 0.122; main effect of time: *F*_(3,27)_ = 0.535, *p* = 0.669; stimulation condition by time interaction: *F*_(9,81)_ = 0.597, *p* = 0.796; left M1, glutamate: main effect of stimulation condition: *F*_(3,27)_ = 1.329, *p* = 0.118; main effect of time: *F*_(3,27)_ = 1.236, *p* = 0.316; stimulation condition by time interaction: *F*_(9,81)_ = 1.131, *p* = 0.351).

### Both anodal and cathodal tDCS to left M1 lead to a significant decrease in GABA in the unstimulated right M1

**Figure 2.**
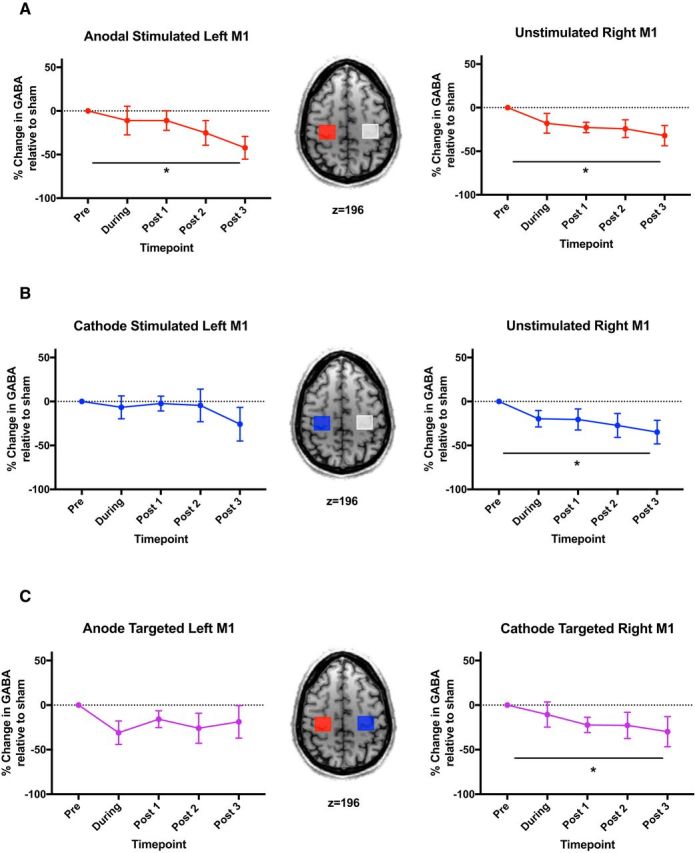
Percentage change in GABA during and after anodal(***A***), cathodal (***B***), and bilateral (***C***) stimulation relative to sham stimulation. Brain images show the location of MRS voxels and stimulation. Red indicates anode placement, blue cathode placement, and gray no electrode placement. *Significant main effect of condition for each sham versus real stimulation comparison of the baselined GABA concentrations. Error bars indicate SEM. ***A***, Decrease in GABA was observed in both stimulated and nonstimulated M1s in response to anodal tDCS (right M1, *n* = 12; left M1, *n* = 9). ***B***, Significant decrease in GABA was observed only within the nonstimulated right M1 in the cathodal tDCS condition (right M1, *n* = 10; left M1, *n* = 7). ***C***, Significant decrease in GABA was observed only within the cathode-targeted right M1 in response to bilateral tDCS condition (right M1, *n* = 10; left M1, *n* = 8).

To investigate the changes in GABA in each hemisphere due to each stimulation protocol directly, we then tested each real stimulation condition against the sham session using RM-ANOVAs with one factor of stimulation condition (real, sham) and one factor of time (during, Post 1, Post 2, Post 3). This approach demonstrated a decrease in GABA concentration in response to anodal tDCS compared with sham in both the stimulated left M1 (main effect of stimulation condition: *F*_(1,8)_ = 8.964, *p* = 0.017, no significant main effect of time: *F*_(3,24)_ = 2.711, *p* = 0.067; no stimulation by time interaction: *F*_(3,24)_ = 0.611, *p* = 0.614) and the nonstimulated right M1 (main effect of stimulation condition: *F*_(1,11)_ = 7.898, *p* = 0.017; no significant effect of time: *F*_(3,33)_ = 1.278, *p* = 0.298; no stimulation by time interaction: *F*_(3,33)_ = 0.816, *p* = 0.494; Fig. 2*A*).

Cathodal tDCS did not modulate GABA within the stimulated left M1 (main effect of stimulation condition: *F*_(1,6)_ = 1.410, *p* = 0.280; no significant main effect of time: *F*_(3,18)_ = 1.102, *p* = 0.374; no stimulation condition by time interaction: *F*_(3,18)_ = 0.517, *p* = 0.676), but there was a significant decrease in GABA within the nonstimulated right M1 (main effect of stimulation condition: *F*_(1,9)_ = 13.106, *p* = 0.006; no significant main effect of time: *F*_(3,27)_ = 1.530, *p* = 0.229; no stimulation condition by time interaction: *F*_(3,27)_ = 0.455, *p* = 0.716; Fig. 2*B*).

Bilateral tDCS did not modulate GABA within the anode-targeted left M1 (main effect of stimulation condition: *F*_(1,7)_ = 0.894, *p* = 0.376; no significant main effect of time: *F*_(3,21)_ = 2.913, *p* = 0.058; no stimulation by time interaction: *F*_(3,21)_ = 0.062, *p* = 0.979), but there was a significant decrease in GABA within the cathode-targeted right M1 (main effect of stimulation condition: *F*_(1,9)_ = 8.058, *p* = 0.019; no significant effect of time: *F*_(3,27)_ = 0.382, *p* = 0.767; no stimulation by time interaction: *F*_(3,27)_ = 2.149, *p* = 0.117; Fig. 2*C*).

### tDCS-related GABA changes in nonstimulated right M1 can be explained by white matter microstructure.

Finally, we wished to investigate how change in GABA in the nonstimulated hemisphere during tDCS might relate to white matter microstructure between the two M1s. We first wished to investigate whether there was a correlation between tDCS-induced GABA change within the two M1s. No significant relationship was demonstrated for any of the stimulation conditions (anodal tDCS: *r*_(11)_ = −0.014, *p* = 0.965; cathodal tDCS: *r*_(9)_ = −0.268, *p* = 0.453; bilateral: *r*_(8)_ = −0.117, *p* = 0.765).

However, given the strong structural connectivity between the two M1s, we then went on to investigate whether tDCS-induced GABA change in the unstimulated M1 was related to the microstructure of white matter tracts connecting the two M1s on a subject-by-subject basis.

**Figure 3.**
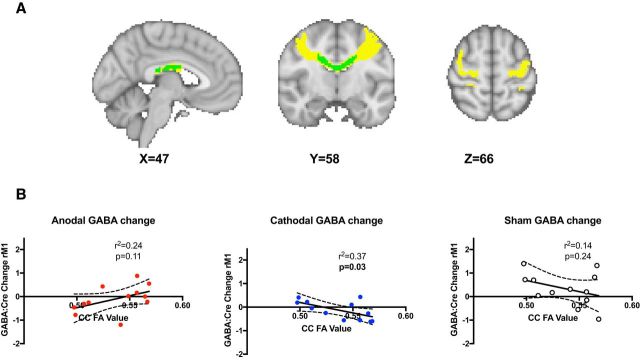
***A***, M1–M1 CC mask. The M1–M1 tract is shown in yellow and the M1–M1 CC in green. ***B***, Cathodal tDCS-induced GABA change within right M1 correlates with FA in the M1–M1 CC mask. No significant relationship between M1–M1 CC FA and change in GABA in right M1 was demonstrated during either anodal or cathodal tDCS.

We demonstrated a significant relationship between the change in GABA during stimulation in the right (unstimulated) M1 and white matter microstructure between the stimulated and nonstimulated M1s, as assessed by FA for cathodal tDCS (*r*_(11)_ = −0.610, *p* = 0.035), but not for anodal tDCS (*r*_(11)_ = 0.487, *p* = 0.108), or in the sham control (*r*_(11)_ = −0.369, *p* = 0.237) (Fig. 3*B*). To assess the anatomical specificity of this result, we investigated the relationship between FA within the two control regions, the section of CC linking left M1 to right somatosensory cortex and the CST and change in GABA levels in right M1 in response to stimulation, and demonstrated no significant correlations (CC: anodal: *r*_(11)_ = 0.435, *p* = 0.158; cathodal: *r*_(11)_ = −0.325, *p* = 0.302; sham: *r*_(11)_ = −0.575, *p* = 0.051; CST: anodal: *r*_(11)_ = 0.160, *p* = 0.620; cathodal: *r*_(11)_ = −0.069, *p* = 0.831; sham: *r*_(11)_ = −0.138, *p* = 0.668). Furthermore, no significant correlation was found for any stimulation condition between changes in glutamate levels in right M1 and FA in either the M1–M1 CC mask (anodal: *r*_(11)_ = 0.382, *p* = 0.221; cathodal: *r*_(11)_ = −0.472, *p* = 0.121; sham: *r*_(11)_ = 0.056, *p* = 0.862) or CST mask (anodal: *r*_(11)_ = −0.096, *p* = 0.767; cathodal: *r*_(11)_ = −0.453, *p* = 0.139; sham: *r*_(11)_ = 0.445, *p* = 0.147).

## Discussion

This study was performed to explore the physiological changes underpinning motor plasticity across the motor network. We chose to use tDCS as our plasticity-induction protocol as it enabled us to directly target one M1 while studying the neurochemical changes in both M1s. We have replicated previous findings (Stagg et al., 2009; Stagg and Nitsche, 2011; Kim et al., 2014; Bachtiar et al., 2015) that anodal tDCS applied to M1 causes a decrease in MRS-measured GABA levels in the stimulated area of cortex, but we have also shown for the first time that this decrease is accompanied by a concurrent decrease in GABA in the nonstimulated M1. Cathodal tDCS led to a significant decrease in GABA in the nonstimulated right M1, though with no significant GABA changes in the cathode-targeted left M1. Bilateral tDCS resulted in a GABA decrease only in the cathode-targeted right M1.

Our findings are in broad agreement with that of O'Shea et al. (2014), who used TMS to examine changes to cortical excitability in both M1s after tDCS, which demonstrated significant increases in corticospinal excitability in both hemispheres following anodal stimulation to left M1, consistent with our finding of a bilateral GABA decrease. The same study found a decrease in corticospinal excitability in the stimulated hemisphere in the cathodal condition, where we found no change in GABA, but an increase in excitability in nonstimulated hemisphere, which is consistent with our finding of a GABA decrease.

For all conditions in which a GABA decrease was observed, the change persisted into the final MRS measurement. This indicates that tDCS-induced changes in neurochemicals endure for at least 30 min after stimulation. This is perhaps unsurprising given that effects of anodal tDCS on corticospinal excitability have been shown to outlast stimulation by up to 90 min (Nitsche and Paulus, 2001). Further studies over longer timescales are therefore required to determine the duration of tDCS-induced GABA changes.

We found no effect of stimulation on glutamate concentration in either hemisphere. A previous study has demonstrated changes in glutamate concentration in the stimulated region with cathodal tDCS to M1 (Stagg et al., 2009), but another failed to find the same effect (Kim et al., 2014). A recent review examined the influence of factors such as field strength, experimental design, and sequence on MRS-measured changes in glutamate (Mullins, 2018); it may be that differences in design and acquisition time of tDCS-MRS studies may be responsible for the differing results. Futhermore, as is common in many tDCS-MRS studies, we had a relatively small sample size. Replicating this paradigm with a larger sample size will be important to allow robust conclusions to be drawn.

### Bilateral decrease in M1 GABA with anodal tDCS to left M1

Anodal tDCS has been shown to induce LTP-like plasticity in M1 in a manner at least similar to the mechanisms underpinning motor learning and recovery after stroke (Bindman et al., 1964; Weiss et al., 1998; Stagg and Nitsche, 2011; Di Lazzaro et al., 2012). Anodal tDCS therefore provided an experimental model with which to explore neurochemical changes across the wider motor network in response to a specific plasticity-induction protocol and has suggested a decrease in GABA as a common mediating factor across the motor network. Consistent with this hypothesis, decreases in GABA in M1 have been demonstrated when individuals practice a motor skill (Floyer-Lea et al., 2006) and tDCS-induced changes in MRS measured GABA within M1 have been shown to correlate with individuals' ability to learn motor tasks (Stagg et al., 2011a; Kim et al., 2014), whereas the same correlations were not seen with MRS-measured glutamate (Kim et al., 2014). These results highlight the potential importance of change in GABA concentration in mediating motor plasticity and examining GABA changes during learning under tDCS would be an important avenue to explore in future studies.

Here, we saw a decrease in GABA in both the anodal stimulated and nonstimulated hemispheres, suggesting for the first time that a decrease in GABA outside of the stimulated M1 may be important for motor plasticity. However, there was no significant relationship between the change in GABA in the nonstimulated hemisphere and the white matter microstructure between the two M1s in the anodal condition. Although this is perhaps a surprising result at first sight, it would be consistent with the hypothesis that the interhemispheric effects of anodal tDCS rely on facilitation between the two M1s. Because the majority of the transcallosal fibers from M1 to M1 project onto local inhibitory interneurons in the hand area (Schnitzler et al., 1996), it is therefore perhaps not surprising that the microstructure of this tract does not relate to GABA change that appears to be caused by interhemispheric facilitation, though this explanation is speculative and requires further testing.

### Magnitude of GABA decrease in unstimulated hemisphere during to cathodal tDCS relates to underlying white matter microstructure

Cathodal tDCS to the contralesional hemisphere has been suggested as a potential adjunct therapy in stroke recovery (Fregni et al., 2005; Boggio et al., 2007; Kim et al., 2010; Stagg et al., 2012). It has been hypothesized that cathodal tDCS improves function via a transcallosally mediated increase in excitatory activity in the contralateral M1 (Fregni et al., 2005; Boggio et al., 2006; Kim et al., 2010; Stagg et al., 2012; O'Shea et al., 2014) because cathodal tDCS has previously been demonstrated to decrease transcallosal inhibition between the motor cortices (Lang et al., 2004; Bradnam et al., 2010; Tazoe et al., 2014).

Here, we provide evidence to support this hypothesis by demonstrating that cathodal tDCS results in a decrease in inhibition in the contralateral M1 and, further, that this effect is positively correlated with the white matter microstructure between the two cortices such that a higher FA value in the CC was linked to a greater decrease in GABA levels within the right, nonstimulated M1. There is a strong positive correlation between an individuals' transcallosal inhibition and the microstructure of the M1–M1 callosal tracts as measured by FA (Wahl et al., 2007; Fling et al., 2013) and the link between greater FA within the CC and increased effect of tDCS in the unstimulated hemisphere is broadly consistent with previous findings showing a link between FA values within transcallosal fibers and tDCS-induced fMRI laterality index during a motor task (Lindenberg et al., 2013). Together, these findings may provide an explanation for some of the variability in response to cathodal tDCS in the stroke recovery literature and more generally (Chew et al., 2015; López-Alonso et al., 2015) and raises a hypothesis to test: that stroke patients with relatively structurally intact callosal fibers (higher FA) may benefit from cathodal tDCS applied to the contralesional M1, whereas those with less structural integrity (lower FA) in their callosal fibers may respond better to other approaches.

For all participants in this study, the stimulation was applied to the dominant hemisphere, so it may not be possible to generalize these findings to individuals who are left-handed or to stimulation of the nondominant hemisphere.

### Physiological effects of bilateral tDCS are not simply a sum of those underpinning anodal and cathodal tDCS

Bilateral tDCS led only to a decrease in GABA in the cathode-targeted M1. Although this finding is somewhat difficult to interpret, it is important to highlight that, consistent with previous studies (O'Shea et al., 2014; Waters et al., 2017), bilateral stimulation cannot simply be explained as the combined effect of the anode and cathode conditions. In the bilateral M1 montage, current flows transversely across the cortex, as opposed to the radial current flow direction in the unilateral montages. Recent work by Rawji et al. (2018) has shown that the orientation of current flow across the motor cortex influences corticospinal excitability, with current flowing orthogonal to the central sulcus eliciting the greatest changes. It has previously been proposed that this difference in current flow direction between the bilateral and unilateral montages may alter the influence of the tDCS polarity on behavioral (Waters et al., 2017) and neurophysiological (O'Shea et al., 2014) metrics, a hypothesis that would be supported by the data presented here. Together, these findings suggest that further data are needed before bilateral tDCS can be fully optimized as a neuroscientifically informed adjunct therapy in stroke recovery.

### Conclusions

These results show for the first time that neurochemical changes are observed outside of the targeted primary motor cortex during plasticity induction in humans, supporting the role of the wider motor network in motor plasticity. The importance of the wider motor network in plasticity is further highlighted by the relationship demonstrated between the anatomically distant effects of cathodal tDCS and the underlying white matter microstructure, findings that, together, begin to explain the putative effectiveness and the well described variability of tDCS in stroke recovery.
